# Monte Carlo derivation of filtered tungsten anode X-ray spectra for dose computation in digital mammography*

**DOI:** 10.1590/0100-3984.2014.0108

**Published:** 2015

**Authors:** Lucas Paixão, Bruno Beraldo Oliveira, Carolina Viloria, Marcio Alves de Oliveira, Maria Helena Araújo Teixeira, Maria do Socorro Nogueira

**Affiliations:** 1 M.Sc., Post-graduation in Science and Technology of Radiations, Minerals and Materials - Centro de Desenvolvimento da Tecnologia Nuclear / Comissão Nacional de Energia Nuclear (CDTN/CNEN), Belo Horizonte, MG, Brazil.; 2 M.Sc., Post-graduation in Nuclear Sciences and Techniques - Departamento de Engenharia Nuclear da Universidade Federal de Minas Gerais (DEN-UFMG), Belo Horizonte, MG, Brazil.; 3 M.Sc., Professor, Department of Anatomy and Imaging, Universidade Federal de Minas Gerais (UFMG), Belo Horizonte, MG, Brazil.; 4 MD, Radiologist, Technical Director, Clínica Dra. Maria Helena Araújo Teixeira, Belo Horizonte, MG, Brazil.; 5 D.Sc., Titular Researcher-Professor, Centro de Desenvolvimento da Tecnologia Nuclear / Comissão Nacional de Energia Nuclear (CDTN/CNEN), Departamento de Engenharia Nuclear da Universidade Federal de Minas Gerais (DEN-UFMG), Belo Horizonte, MG, Brazil.

**Keywords:** Mammography, X-ray spectra, HVL, Monte Carlo

## Abstract

**Objective:**

Derive filtered tungsten X-ray spectra used in digital mammography systems by means of Monte Carlo simulations.

**Materials and Methods:**

Filtered spectra for rhodium filter were obtained for tube potentials between 26 and 32 kV. The half-value layer (HVL) of simulated filtered spectra were compared with those obtained experimentally with a solid state detector Unfors model 8202031-H Xi R/F & MAM Detector Platinum and 8201023-C Xi Base unit Platinum Plus w mAs in a Hologic Selenia Dimensions system using a direct radiography mode.

**Results:**

Calculated HVL values showed good agreement as compared with those obtained experimentally. The greatest relative difference between the Monte Carlo calculated HVL values and experimental HVL values was 4%.

**Conclusion:**

The results show that the filtered tungsten anode X-ray spectra and the EGSnrc Monte Carlo code can be used for mean glandular dose determination in mammography.

## Abstract (pt)

**Objetivo:**

Derivar espectros filtrados de raios X de tungstênio utilizados em sistemas de mamografia digital por meio de simulações Monte Carlo.

**Materiais e Métodos:**

Espectros filtrados por filtro de ródio foram obtidos para potenciais do tubo entre 26 e 32 kV. Os valores de camada semirredutora (CSR) dos espectros filtrados simulados foram comparados aos valores obtidos experimentalmente com um detector de estado sólido Unfors modelo 8202031-H Xi R/F & MAM Detector Platinum e unidade base 8201023-C Xi Platinum Plus w mAs em um sistema Hologic Selenia Dimensions utilizado no modo radiografia direta.

**Resultados:**

Os valores de CSR calculados mostraram boa concordância quando comparados com os valores obtidos experimentalmente. A maior diferença relativa entre os valores de CSR calculados com Monte Carlo e os valores de CSR experimentais foi 4%.

**Conclusão:**

Os resultados demonstram que os espectros filtrados de raios X de ânodo de tungstênio e o código de Monte Carlo EGSnrc podem ser utilizados para determinar a dose glandular média em mamografia.

## INTRODUCTION

Radiographic breast imaging (mammography) is indicated for detection, diagnosis and clinical management of cancer. Moreover, mammography is the most widely used imaging modality for breast cancer screening^(1)^. Breast dosimetry is an important part of the quality assurance program, provides means to define and verify the standards of good practice, besides contributing in the optimization of radiological protection^(2,3)^.

It is widely accepted that the mean glandular dose (*D_G_*) for the breast glandular tissue is the most useful magnitude for characterizing breast cancer risk^(2,4)^. Because of the difficulty to measure it directly on the breast, the procedure to estimate the DG values consists in making use of conversion factors that relate incident air kerma (*K_i_*) at this dose value. Generally, the conversion factors vary with the X-ray spectrum half-value layer (HVL) and the breast composition and thickness. By means of computer simulations, several authors have calculated such factors with the Monte Carlo method^(5-8)^. The Monte Carlo radiation transport simulations are recognized as an important tool in dose calculations in different fields related to medical physics^(9)^. Monte Carlo codes can be used in mammography to simulate and characterize photon beams produced; radiation dose absorbed by patient's organs; and dosimetry involving phantoms^(10)^.

Many X-ray spectral models for *D_G_* computer simulations purposes are available in the diagnostic range^(11,12)^. One of the models available^(13)^ generates polyenergetic X-ray spectra for molybdenum, rhodium, and tungsten anodes. The spectra produced by this model do not include any added filtration except by the 0.5 mm beryllium window of the X-ray tube and any self-filtration by the anode itself.

The objective of the present study is to use Monte Carlo simulations to generate filtered X-ray spectra used in digital mammography systems from unfiltered spectra. Therefore, the Monte Carlo EGSnrc code package with the C++ class library (egspp) was employed to derive filtered tungsten X-ray spectra. Filtered spectra for rhodium filter were obtained for tube potentials between 26 and 32 kV. The HVLs of simulated filtered spectra were compared with those experimentally obtained with a solid state detector in a digital mammography system to validate the results. The results were also compared with the values recommended by the Technical Reports Series no. 457 of the International Atomic Energy Agency^(14)^.

## MATERIALS AND METHODS

### Geometric model

**Figure 1:**
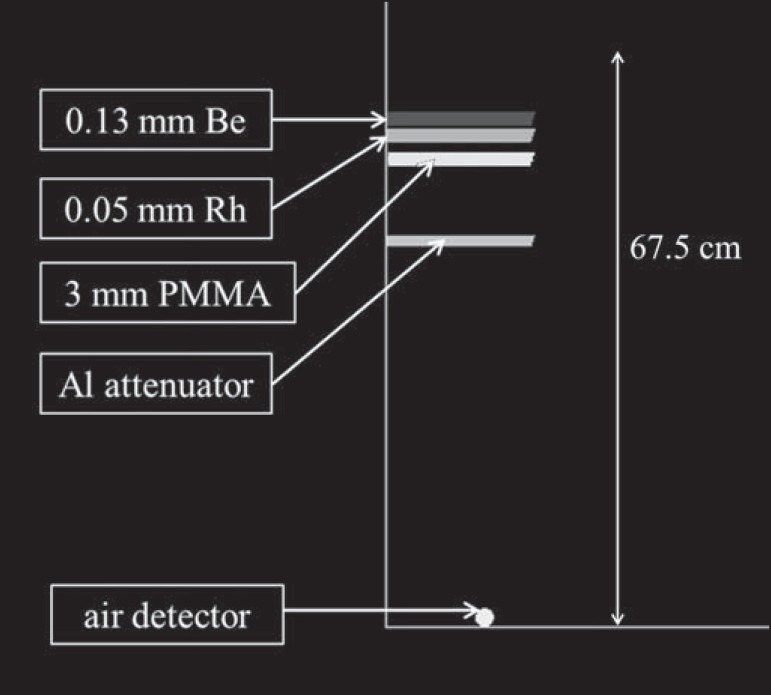
Geometric model schematic drawing.

The geometric model adopted in the simulations was based on the Hologic Selenia Dimensions system (Hologic, Inc.; Bedford, MA) of a mammography clinic located in Belo Horizonte, MG, Brazil. A schematic drawing is shown on Figure 1.

Such a mammography system has a focal spot of 0.3 mm, a 0.63 mm beryllium window and 0.050 mm rhodium filter. The distance between the breast support and the focal spot is 67.5 cm.

Since the spectra have a 0.5 mm beryllium inherent filtration, to simulate the 0.63 mm beryllium window of clinical system, a 0.13 mm beryllium (*ρ* = 1.848 gcm^-3^) window was positioned at 5.0 cm from the focal spot in the simulations. The rhodium (*ρ* = 12.41 gcm^-3^) filter was modelled at 7 cm. Since the HVL measurements are performed in the presence of the compression plate, a polymethylmethacrylate (PMMA) compression plate was modelled with 18.0 × times; 24.0 × 0.3 cm^3^ at 10 cm^(14)^. The aluminium (*ρ* = 2.6989 gcm^-3^) attenuators were positioned at 20 cm, with thicknesses ranging from 0.4 and 0.8 mm. All distances are relative to focal spot. An air (*ρ* = 0.00120479 gcm^-3^) sphere of 6 cm^3^ was centred laterally at 66.37 cm from the tube focal spot and at 6 cm from the chest wall edge as a detector. The breast support and X-ray scatter reduction grid were not modelled. The density and composition of materials utilized in the simulations were obtained in the material library of the EGSnrc code.

### Monte Carlo simulations

**Figure 2:**
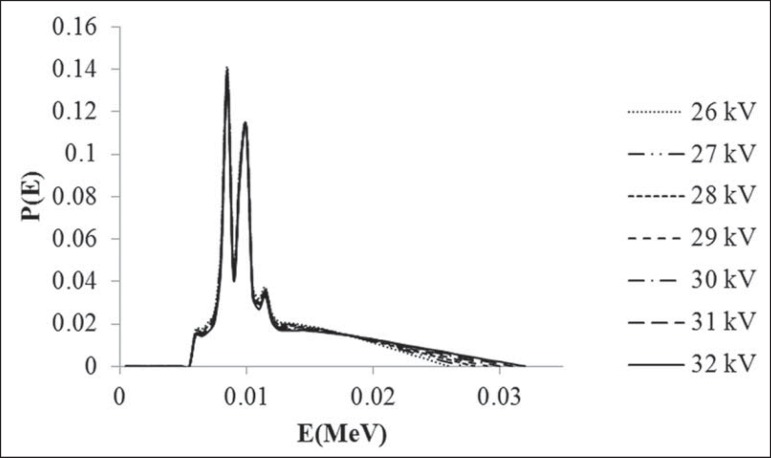
Unfiltered tungsten X-ray spectra used in the Monte Carlo simulations.

The Monte Carlo EGSnrc code package^(15)^ with the C++ class library (egspp) was employed^(16)^. The unfiltered tungsten X-ray spectra for tube potentials between 26 and 32 kV (Figure 2) were simulated^(13)^.

The radiation beam was collimated into a rectangular shape of 1.13 cm side. Thus the collimated beam impinges directly on the modelled detector. Electrons and photons are followed down to a threshold energy of 10 keV. Bound Compton scattering, Electron Impact Ionization, Rayleigh scattering and atomic relaxations are turned on. NIST tabulations of differential bremsstrahlung cross sections and photon cross sections from XCOM tabulations are used^(17)^. Absorbed dose in air simulations were performed for HVL calculations with 5 × 10^7^ histories which represent a statistical error of about 3%. The simulations were performed in a personal computer with an Intel^®^ Xeon^®^ Quad CPU of 3.30 GHz with 4 GB RAM.

### HVL measurements

**Figure 3:**
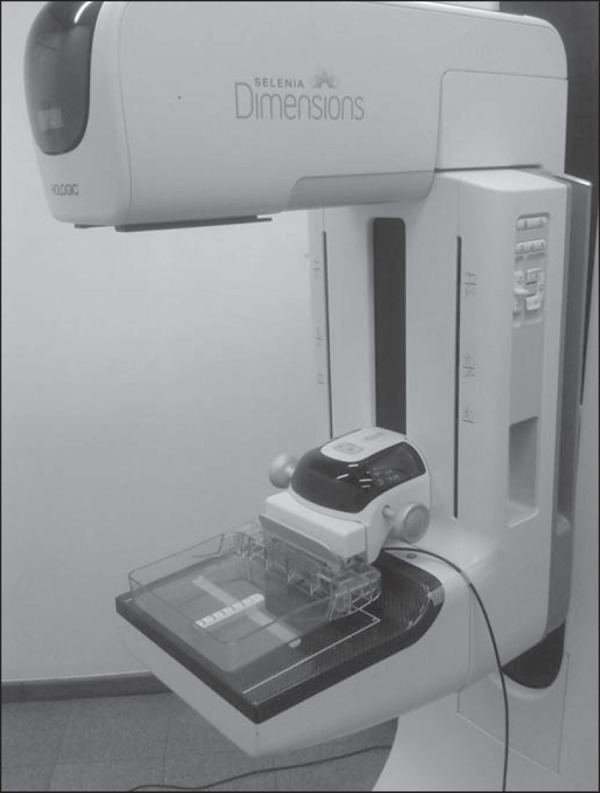
Experimental setup showing the compression plate in contact with detector.

Irradiations were carried out using the W/Rh target/filter combination and a Selenia Dimensions model Hologic DBT system using a direct radiography mode. Measurements were performed using a calibrated set manufactured by Unfors, composed of the solid state detector model 8202031-H Xi R/F & MAM Detector Platinum Series 181096, connected to the base unit model 8201023-C Xi Base unit Platinum Plus w mAs, Series 190046. The solid state detector sensitive volume was laterally centered at 65 cm from the tube focal spot and at 6 cm from the chest wall edge. The X-ray tube voltage was varied from 27 to 31 kV at intervals of 1 kV. The HVL values using the solid state detector were obtained directly by averaging three measurements in mmAl. All irradiations were done with a tube loading of 50 mAs. It is important to observe that irradiations were performed with the compression plate in contact with the detector (Figure 3). This was necessary for future use of the HVL values in the determination of *D_G_* conversion factors^(6,18)^.

## RESULTS

**Figure 4:**
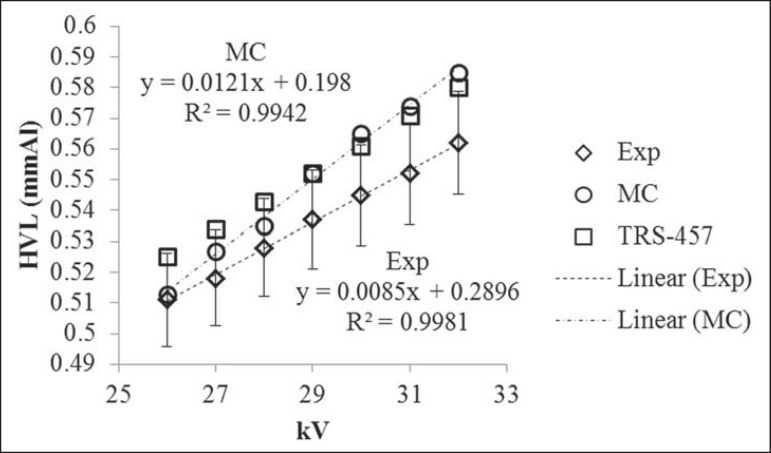
HVL values for each voltage and W/Rh target/filter combination.

The W/Rh target/filter combination X-ray spectra HVL values obtained by Monte Carlo simulations and experimentally are shown on Table 1. The experimental values for 26 and 32 kV were estimated from linear fitting of the remaining values. The uncertainty of all results was estimated to be 3% (1 σ). The estimated values uncertainty was < 3% but this value was maintained in order to be conservative. The experimental HVL values uncertainty calculation is shown on Table 2. The main sources of experimental values uncertainty are calibration (2%) and energy dependence (2%) of the detector. The HVL values obtained in the present study are also shown on Figure 4, along with HVL values recommended by TRS-457^(14)^. Linear fitting of the data along with its equation and the R^2^ coefficient are also shown on Figure 4. For a better viewing, only experimental values uncertainty bars are shown.

**Table 1:** HVL values for each voltage and W/Rh target/filter combination.

HVL (mmAl)		
kV	Experimental	Monte Carlo
26	0.511 ± 3%*	0.513 ± 3%
27	0.518 ± 3%	0.527 ± 3%
28	0.528 ± 3%	0.535 ± 3%
29	0.537 ± 3%	0.552 ± 3%
30	0.545 ± 3%	0.565 ± 3%
31	0.552 ± 3%	0.574 ± 3%
32	0.562 ± 3%*	0.585 ± 3%

* Estimated values.

**Table 2:** Experimental HVL values uncertainty calculation.

Uncertainty sources	Uncertainty (%)
Calibration	2.0
Detector resolution	0.5
Angular dependence	0.5
Temperature and humidity	0.5
Energy dependence	2.0
Readings accuracy*	0.3
Measurement position†	0.3
Combined standard uncertainty (k = 1)	3.0

* Standard deviation of the average of three measurements.

† ± 2 mm for 700 mm.

## DISCUSSION

Calculated HVL values showed good agreement as compared with those experimentally obtained. The highest relative percentage difference between the Monte Carlo calculated HVL values and experimental HVL values was 4%. Taking into account the values recommended by the TRS-457, the relative percentage difference for Monte Carlo calculated HVL values ranged between -2% and 1% and was -3% for all experimental values. Such Monte Carlo calculated HVL values are preliminary results. Great improvement has been achieved for both results and uncertainties by using *K_i_* rather than absorbed dose in air. New results are reported by Paixão et al.^(19)^.

**Figure 5:**
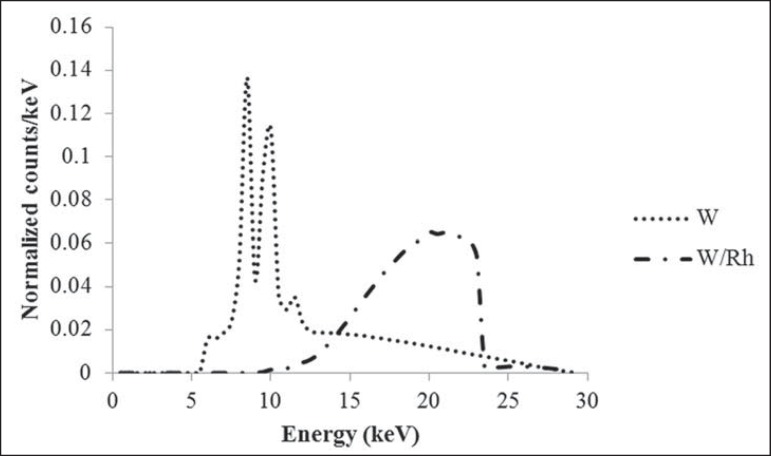
Tungsten target X-ray spectra^(13)^ and Monte Carlo obtained W/Rh target/filter combination X-ray spectra for 29 kV.

The results obtained in this study show that the EGSnrc Monte Carlo code generates the X-ray spectra with appropriate filtration. As an example, a Monte Carlo obtained W/Rh target/filter combination X-ray spectra for 29 kV is shown on Figure 5. The filtered tungsten anode X-ray spectra may be used for dosimetry studies in mammography. When using the filtered spectrum instead of the unfiltered spectrum in dose simulations, one may gain in computational time.

Although Mo/Mo target/filter is the most widely used combination in clinical practice, new combinations have been introduced with the increasing use of digital mammography systems^(8)^. Therefore, the results of the present study are important as they can be applied to state of the art equipment.

## CONCLUSIONS

In the present study, the Monte Carlo code EGSnrc was employed for simulation of filtered X-ray spectra used in digital mammography. The differences in the HVL values were less than 4% for all tube voltages. Such results demonstrate that the EGSnrc code provides a filtration of the raw X-ray spectra in good agreement with those experimentally determined. The W/Rh target/filter combination X-ray spectra obtained in the simulations may be used in future Monte Carlo simulations studies in digital mammography.
